# Electromyographic Patterns of Muscle Activation During Running with Different Footwear at Different Speeds in Nulliparous Women: A Secondary Analysis

**DOI:** 10.3390/s25103016

**Published:** 2025-05-10

**Authors:** María García-Arrabé, Fabien Guerineau, Beatriz Ruiz-Ruiz, Javier López-Ruiz, Mónica García-Mateos, María-José Giménez

**Affiliations:** Department of Physiotherapy, Faculty of Medicine, Health and Sports, European University of Madrid, 28670 Villaviciosa de Odón, Madrid, Spain; maria.gararrabe@universidadeuropea.es (M.G.-A.); fabien.guerineau@universidadeuropea.es (F.G.); beatriz.ruiz@universidadeuropea.es (B.R.-R.); javier.lopez3@universidadeuropea.es (J.L.-R.); monica.garcia@universidadeuropea.es (M.G.-M.)

**Keywords:** electromyography, pelvic floor, minimalist shoes, core stability, female runners

## Abstract

With the global increase in women’s participation in running, understanding factors like footwear in performance and injury prevention has become essential. Minimalist shoes (MSs) and traditional shoes (TSs) influence muscle activation patterns, affecting running technique. Proper coordination of the core muscles is essential for efficient stride and posture. This study analyzed muscle activation in nulliparous women running in MSs and TSs at different speeds and explored the correlations with age and BMI. A crossover clinical trial assessed the EMG activation of the lumbar erector (LE), gluteus maximus (GM), pelvic floor, and internal oblique (IO) muscles during treadmill running at 6, 9, and 11 km/h. Fifty-one healthy women (26.55 ± 5.11 years; body mass index (BMI): 21.29 ± 2.07 kg/m^2^) participated. The protocol included a warm-up, 30 s runs at each speed, and a 5-minute washout between trials. The statistical analyses included Wilcoxon, Friedman, and Spearman’s correlation tests. GM and IO showed the highest activation (*p* < 0.001) regardless of the footwear or speed. No significant differences were found between MSs and TSs. Weak-to-moderate correlations emerged between BMI and LE muscle activation with MSs, and between BMI and IO with both footwear. Significant correlations were also found with IO activations, but none with PF muscles. The correlations between personal variables, shoe types, and muscle activation suggest that individual and external factors may influence neuromuscular modulation, impacting injury prevention and personalized interventions.

## 1. Introduction

Running has become one of the most popular physical activities in recent decades, particularly among women [1]. This increase in female participation has led to greater interest in understanding the biomechanical and physiological factors that affect female runners, as well as the elements that influence their performance and injury prevention [2].

One of the most relevant concerns is pelvic floor (PF) injuries, a condition that significantly affects female runners due to specific anatomical, hormonal, and biomechanical factors [3]. The PF, a structure composed of muscles, ligaments, and connective tissue, is essential for the stability of the abdominopelvic region, also known as the core, and for intra-abdominal pressure control [4]. In female runners, the repetitive load associated with running impact may contribute to PF dysfunctions, such as urinary incontinence or prolapse [5].

Meanwhile, core muscles, including the transverse abdominis, internal and external obliques, rectus abdominis, lumbar erector spinae (LE) muscle, and gluteus maximus (GM), play a crucial role in trunk stabilization during running [6]. Proper activation of these muscles not only enhances biomechanical efficiency but also protects PF structures by distributing loads more evenly [7].

During running, adequate activation of the abdominopelvic region is essential for maintaining an efficient posture and reducing injury risk. Insufficient or uncoordinated core muscle activation may lead to excessive strain on the PF, increasing the risk of the aforementioned dysfunctions [8].

In this context, running footwear plays a crucial role, not only in running efficiency but also in injury incidence, particularly those related to the PF [9]. Currently, there is an ongoing debate regarding the differences between traditional and barefoot footwear and their impacts on PF health and muscle activation during running [10].

Barefoot footwear, characterized by its minimalist design, intended to mimic the natural biomechanics of barefoot running, has gained popularity as an alternative to traditional footwear. While traditional shoes (TSs) provide greater cushioning and support, minimalist shoes (MSs) promote a more natural foot strike pattern and increased activation of intrinsic foot muscles [11]. However, the relationship between footwear type and PF injuries in female runners remains insufficiently explored. Recent studies suggest that footwear choice may influence the magnitude of impact forces transmitted to the PF [12]. While traditional footwear provides substantial cushioning, MSs designed to reduce impact through energy absorption mechanisms could represent a promising alternative for minimizing these forces [13]. Nevertheless, their effect on muscle activation and core stability is still not fully understood [14].

Electromyographic (EMG) studies have shown that abdominopelvic muscle activation varies depending on the type of footwear used, suggesting that footwear selection may influence a runner’s ability to maintain a stable posture and reduce stress on the PF [15]. Traditional footwear, with greater cushioning and support, tends to encourage a heel-strike pattern, which may reduce impact absorption by core muscles [16]. In contrast, barefoot footwear promotes a forefoot-strike pattern, associated with greater activation of intrinsic foot and lower leg muscles, leading to increased stabilization demands on the abdominopelvic region [17]. This heightened activation may have a protective effect on the PF by better distributing impact forces and reducing stress on this structure [18,19].

A study conducted by our group analyzed the EMG activity of abdominal lumbopelvic muscles in healthy, nulliparous women during running at different speeds (9, 11, and 13 km/h) and with different shoes: MSs and TSs [20]. However, to date, the specific distribution and activation patterns of the abdominopelvic muscles, the PF, LE, internal obliques (IO), and GM, as well as the correlation between these activation patterns and various sociodemographic characteristics, remain unexplored. EMG analysis enables the real-time visualization of muscle activation patterns, allowing for the identification of muscle synergies and coactivation strategies, thus providing insights into normative muscular behaviors during running. Therefore, further investigation into the distribution of muscle activation is necessary.

In this context, the present analysis aimed to analyze the distribution and activation patterns of the abdominopelvic musculature using traditional and minimalist footwear at three running speeds (6, 9, and 11 km/h). We hypothesized that minimalist footwear induces greater activation of the selected core muscles compared with traditional footwear and that significant correlations exist between muscle activation levels and specific sociodemographic variables.

Through this biomechanical analysis, the present study aimed to provide practical recommendations for footwear selection and injury prevention in female runners.

## 2. Materials and Methods

### 2.1. Study Design

A crossover clinical trial was designed to evaluate the impact of minimalist and traditional footwear on the EMG and biomechanical activity of healthy, nulliparous women during treadmill running at three speeds (6, 9, and 11 km/h). Participants were randomized into two groups to determine the order of footwear usage.

Randomization was conducted in a blinded manner by an external team member using a random permutation table. The assignment for each participant was recorded in sealed cards, which were opened by the principal investigator at the start of the intervention.

The interventions were carried out at the Radiology, Rehabilitation, and Physiotherapy Department of the Faculty of Nursing, Physiotherapy, and Podiatry at Complutense University of Madrid. This study was approved by the Research and Ethics Committee of the Hospital Clínico San Carlos (Code 19/570-E_TFM) and adhered to the principles of the Declaration of Helsinki. All participants signed a written informed consent form. Additionally, the trial was registered at ClinicalTrials.gov (CI: NCT04457141) and followed the CONSORT guidelines.

### 2.2. Study Subjects

This study included healthy, nulliparous women who met the following inclusion criteria: aged between 18 and 38 years, nulliparous, clinically healthy at the time of this study, body mass index (BMI) ≤ 30 kg/m^2^, and habitual users of traditional running shoes for sports activities.

The inclusion of nulliparous women aimed to avoid potential pelvic floor dysfunctions or biomechanical alterations resulting from pregnancy and childbirth, factors that could introduce bias and confound the assessment of this study’s outcomes.

The exclusion criteria included pregnancy, urogenital dysfunction, recent lower-limb surgeries, red or yellow flags at the lumbopelvic level (e.g., urinary incontinence, surgeries, tumors, and endometriosis), and an inability to perform voluntary PF contractions, as assessed by the Oxford Scale. The sample was recruited through advertisements in university centers in Madrid and social networks.

### 2.3. Footwear Types

**Figure 1:**
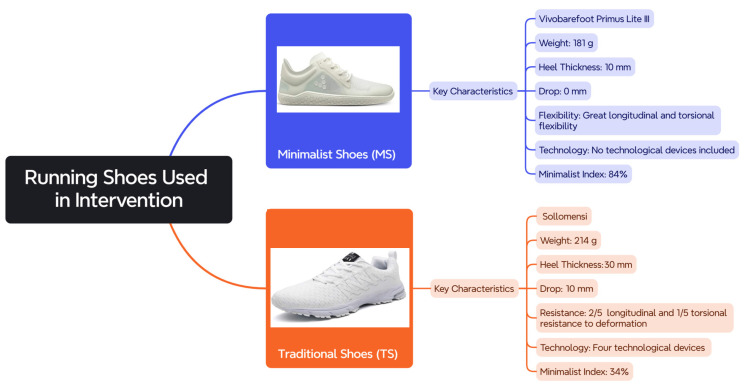
Key characteristics of the two types of footwear used during this study.

Participants used the Vivobarefoot Primus Lite III (Vivobarefoot, London, UK) as MSs and the Sollomensi model (Sollomensi, Beijing, China) as TSs. The characteristics of both types of footwear (weight, sole thickness, drop, length, and torsional flexibility technology) are detailed in Figure 1, and their minimalist index (MI) was calculated according to the scale developed by Esculier et al. [11].

These specific models were selected because they clearly represent the defining features of minimalist and traditional running shoes, respectively, allowing for a more accurate comparison between the two footwear categories and ensuring consistency with the conceptual distinctions made in the literature.

### 2.4. Electromyographic Signal Acquisition and Processing

Anthropometric data were obtained using a Detecto scale and a Holtain stadiometer. EMG activity was recorded with the mDurance^®^ system (mDurance Solutions SL, Granada, Spain), employing bipolar Shimmer sensors (Shimmer Research Ltd., Dublin, Ireland) to assess four muscles: LE, GM, IO, and PF. These sensors sampled EMG signals at a frequency of 1024 Hz, following the recommendations of the SENIAM project to capture muscle activity during dynamic tasks.

To improve the accuracy of the subsequent EMG analyses during each participant’s treadmill run, the signals were subjected to meticulous filtering procedures. A fourth-order Butterworth bandpass filter with cutoff frequencies set at 20 Hz and 450 Hz was applied to attenuate motion artefacts and high-frequency noise.

After filtering, the EMG signals were rectified and smoothed using a root-mean-square (RMS) method with a window size of 0.025 s and a 50% overlap (0.0125 s). RMS was the primary variable used to represent muscle activity, measured in microvolts (µV).

For normalization, the EMG data were scaled relative to each participant’s maximum voluntary isometric contraction (MVIC). This normalization facilitated comparisons between individuals and experimental conditions.

All processing steps were performed using the mDurance cloud-based platform, ensuring standardized and reproducible analysis across the datasets. The data were recorded and analyzed via the mDurance^®^ application on an Android Galaxy A7 tablet, with cloud-based storage and processing.

### 2.5. Study Protocol

**Figure 2:**
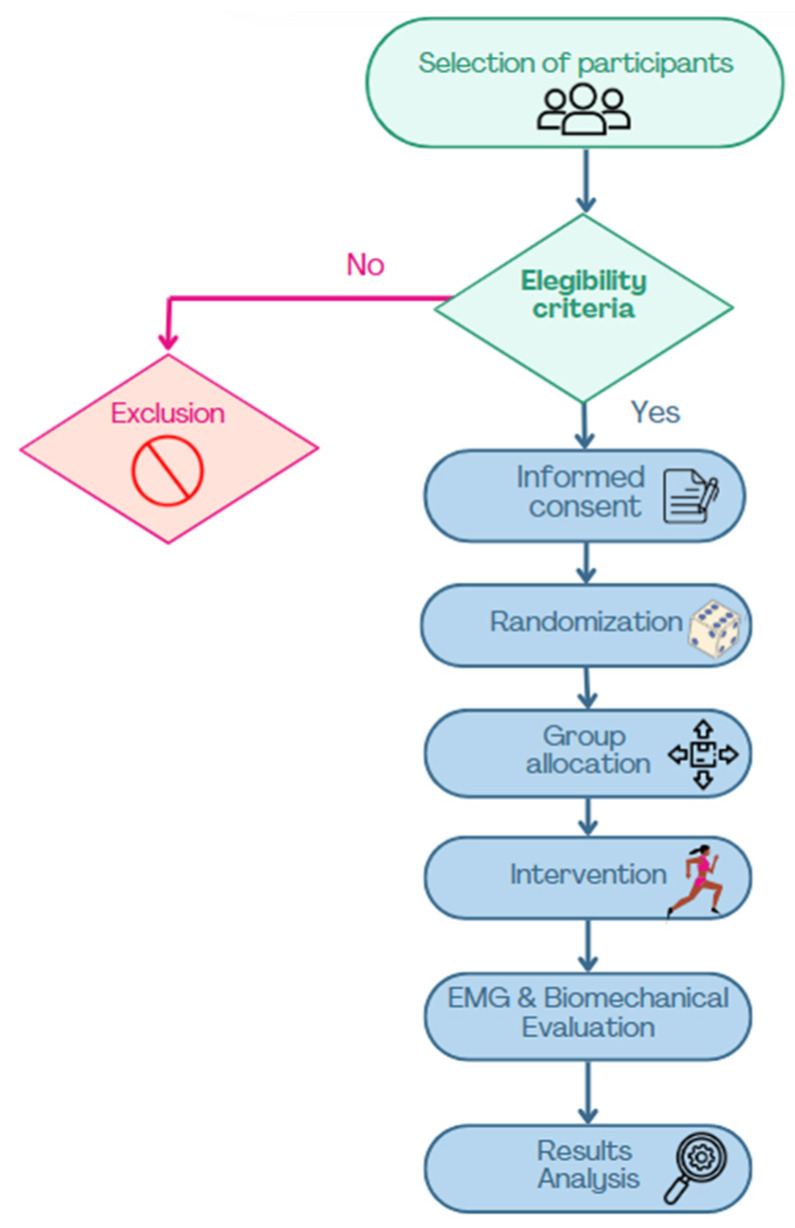
Flowchart of participant selection and study procedures.

Each participant attended a single session. Initially, their age, weight, height, and BMI were recorded. Subsequently, the assigned footwear type was donned, and the protocol commenced on an HP Cosmos Mercury treadmill (Ref. cos 30000va08, HP Cosmos Sport & Medical, Nussdorf-Traunstein, Germany). The protocol included a 5-minute warm-up at a self-paced speed, followed by a testing phase: 30 s at 6 km/h, 30 s at 9 km/h, and 30 s at 11 km/h. A five-minute seated washout period was implemented between trials for each footwear type (Figure 2).

### 2.6. EMG Evaluation

**Figure 3:**
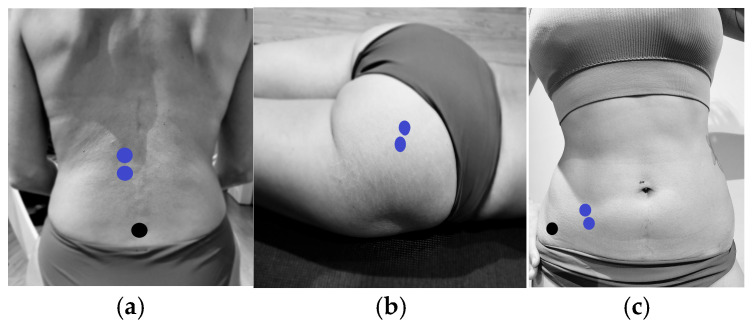
Electrode placement for EMG measurement of lumbar erector spinae, gluteus maximus, and internal oblique muscles. (**a**) Placement of electrodes (blue) on the lumbar erector spinae and reference electrodes (black). (**b**) Placement of electrodes (blue) on the gluteus maximus. (**c**) Placement of electrodes (blue) on the internal oblique and reference electrodes (black).

Skin preparation was performed according to Hermens and Fredriks’ recommendations [21]. For PF recording, the Periform™ intravaginal probe was used, while surface Ag/AgCl electrodes were placed in specific locations for the LE [22], GM [23], and IO [24] (Figure 3):

- LE: Two electrodes were placed longitudinally 3 cm apart, lateral to the spinous process of vertebra L1.
- GM: Two electrodes were placed 2 cm lateral to the mid-sacral crest in a longitudinal arrangement.
- IO: Two electrodes were positioned within the triangle formed by the inguinal ligament, the anterior superior iliac spine (ASIS), and the umbilical midline.

### 2.7. Electromyographic Data Analysis

Prior to the running protocol, the baseline electromyographic (EMG) activity was recorded during a 2 s standing rest period, in which participants maintained a static and relaxed posture. This initial measurement served as a reference point to assess the muscle activation during subsequent dynamic activity.

Maximum voluntary isometric contractions (MVICs) were also recorded for each target muscle. Participants performed three 10 s isometric contractions per muscle, with 20 s of rest between repetitions. The procedures for each MVIC test were as follows:

Internal oblique (IO): Participants lay in a supine position with their knees flexed and were instructed to perform a trunk curl with homolateral rotation.

Gluteus maximus (GM): In a prone position with the right leg flexed, participants executed a hip extension by lifting their thigh off the table.

Lumbar erector (LE): Participants lay prone with their lower legs flexed and performed a trunk extension.

Pelvic floor muscles (PF): Participants were placed in a supine position with their knees bent at 90 degrees.

To ensure the precision of the EMG data collected during treadmill running, the signals were subjected to a comprehensive filtering process. A high-pass Butterworth filter with a cutoff frequency of 20 Hz was applied to eliminate low-frequency motion artefacts, while a low-pass filter with a 450 Hz cutoff was used to reduce high-frequency noise. These settings were selected to isolate the frequency range most relevant to muscle activity during running.

Additionally, a visual inspection of the raw EMG signals was performed prior to the analysis in order to detect and exclude potential artefacts not adequately removed by filtering, such as signal dropouts or movement-induced spikes. This step ensured the reliability and cleanliness of the dataset.

After filtering, the EMG signals were rectified using the mDurance^®^ software (Version 1) via full-wave rectification, converting all signal values into positive values to generate a unipolar waveform. The rectified signals were then smoothed using the root-mean-square (RMS) method over a defined time interval. A window size of 0.025 s with 50% overlap was selected, in line with standard EMG practices. This window length was chosen to balance the temporal resolution and signal stability, enabling the capture of rapid muscle activation changes during running. The RMS was used to normalize the signal, enabling comparisons between the participants and running conditions. The normalized EMG data were expressed as a percentage of each participant’s MVIC.

For each running speed (6, 9, and 11 km/h), we analyzed a 30 s segment of steady-state activity. From this segment, we computed the average muscle activation, as well as the activation ratio of the four evaluated muscles for each speed and footwear type. This multi-step approach ensured rigorous and standardized data processing, providing detailed insights into muscle activation patterns during treadmill running.

### 2.8. Statistical Analysis

The sample size was determined based on convenience, referencing the only previous study by Leitner et al. [25], which assessed the PF during running at different speeds and included 50 participants. Considering a potential 10% dropout rate, a total of 51 participants were selected.

Since in previous analyses, no differences were observed according to the assigned group based on the order of use of the two types of footwear [20,26], for the present analysis, the differences in order were not taken into account, and two matched groups of 51 women each (the MS and TS groups) were analyzed.

Descriptive analysis of the EMG activation distribution during the intervention was performed by the calculation of the median (P_25_, P_75_) values for each muscle with each type of running shoes. Comparisons of the EMG activation of each muscle between groups (MSs and TSs) were performed by the Wilcoxon signed-rank test due to the non-normal distribution of the variables and the paired nature of the data. The Friedman test was used for the comparison of the EMG activations of the different muscles within each type of shoe at each speed. Post hoc analyses were performed using the Wilcoxon pairwise signed-rank test with a Bonferroni correction for multiple comparisons. The correlations between EMG activations and the anthropometric variables of age or BMI were explored using the Spearman rho correlation test. The results were interpreted by applying previously published criteria: 0.0 to 0.3—negligible, 0.3 to 0.5—low, 0.5 to 0.7—moderate, 0.7 to 0.9—high, and 0.9 to 1.0—very high [27].

## 3. Results

A total of 51 women were included in this study. The mean ± SD (range) age was 26.55 ± 5.11 (20.0–38.0) years, weight was 58.24 ± 7.06 (45.0–82.0) kg, height was 1.65 ± 0.06 (1.53–1.82) m, and BMI was 21.29 ± 2.07 (17.28–27.40) kg/m^2^.

Table 1 shows the comparisons of the distribution of EMG activations by muscle and speed. Within each running test, the distribution of the activation among the different muscles was statistically (*p* < 0.001) different, regardless of the type of footwear and speed, the GM and IO muscles always being those showing higher activation. When each muscle was analyzed separately, no significant differences were shown when comparing activations running with both types of shoes at each speed.

No significant correlations were found between age and EMG activations. Weak correlations were found between BMI and LE activation when running with MSs but not with TSs. Significant correlations were also found with IO activations, but none with PF muscles. Table 2 details, by muscle and speed, the significant correlations found between BMI and EMG activations in this study.

## 4. Discussion

The muscle recruitment patterns of the IO, LE, PF, and GM were analyzed during running at three speeds (6, 9, and 11 km/h). The results show that the activation distribution of these four muscle groups follows specific patterns for each muscle, with no significant differences observed across speeds or footwear conditions. This aligns with known activation patterns observed in different exercises and activities (back squat and barbell hip thrust exercises [28]). Given that the participants were healthy women, this pattern may be considered physiological. However, GM and IO exhibited greater activation than the other muscles in all conditions, suggesting their essential role in lumbopelvic stabilization and dynamic trunk control during running functions critical for an efficient and safe technique [29]. This finding is consistent with studies linking weakness or disorganized activation of these muscles with lumbopelvic pain [29].

The coactivation of GM, a powerful hip extensor, and IO, a deep trunk stabilizer and rotator, underscores the functional balance between muscles with opposing yet complementary roles. This synergy is similar to the interaction between PF and IO, as demonstrated in the study by García et al. [18]. Such coactivation may represent an active stabilization strategy for the lumbopelvic complex, contributing to center-of-mass control and protecting passive structures from repetitive loading [30,31].

PF activation remained consistent but moderate compared with the GM and IO. However, its role as a deep stabilizer and modulator of intra-abdominal pressure is crucial, particularly for the efficient transmission of forces from the trunk to the lower limbs [18]. The coordination between PF, IO, and GM suggests an integrated motor control pattern that supports structural integrity during running.

In contrast, the LE exhibited lower activation, aligning with previous studies on healthy female runners [20]. This may be beneficial from a neuromuscular efficiency perspective, as excessive LE recruitment, particularly at higher speeds, could increase vertebral compression, muscular fatigue, and lumbar overload risk [32]. Controlled LE activation, in synergy with deep stabilizers, promotes a more efficient and protective recruitment pattern. However, this normal activation pattern may be altered by pain and various musculoskeletal conditions [22].

From a clinical perspective, these findings highlight the importance of identifying abnormal muscle activation patterns that may limit performance, motor efficiency, or increase injury risk [33]. Altered activation could be associated with an inadequate running technique, persistent pain, previous injuries, or muscular imbalances. EMG analysis facilitates the development of individualized intervention strategies, including motor pattern retraining, the strengthening of deficient muscles, and lumbopelvic stability training [34].

Additionally, the results emphasize the influence of external variables, such as footwear type and BMI, on muscle activation. In our study, we found a positive correlation of BMI with a higher electromyographic activation of the LE during running in minimalist versus traditional shoes, at all speeds. There was also a positive correlation of BMI with greater GM activation during running at 6 km/h with minimalist shoes compared with traditional shoes, as well as a positive correlation between BMI and OI activation at all running speeds and with both shoes. The correlations between BMI and LE/IO activity suggest that a higher body mass leads to greater impact forces. Although in our study, participants did not exceed a 30 kg/m^2^ BMI, within this BMI range, the correlations were consistent with results obtained in previous studies that linked higher BMI values to changes in muscle activation patterns [35], aligning with our findings. This greater activation of the OI with both shoes could be due to the fact that it is a deeper muscle, where surface electromyography may not be able to properly target the muscle activation due to the noise produced by the fat accumulation in the lower abdominal region. However, the LE is not surrounded by so much fat [36], which can more reliably show the effect of the shoes on muscle activation [37,38]. In this context, minimalist footwear, by enhancing proprioception [39,40], promotes an anticipatory neuromuscular response, increasing activation to absorb impact forces [41]. Conversely, conventional footwear, with greater cushioning, may reduce the sensory perception of impact associated with a higher BMI [35], potentially limiting proactive muscle responses and increasing the risk of unanticipated joint loading [42].

These findings reinforce the need for an individualized approach to injury prevention and performance optimization in runners, considering not only biomechanical and anthropometric aspects but also footwear type and its interaction with neuromuscular control.

## 5. Clinical Applications

These findings suggest potential guidance for physiotherapists and athletic trainers aiming to optimize performance and reduce injury risk in female runners. The observed consistent activation of the GM and IO muscles may indicate that these muscles could be considered as priority targets in strength and motor control training programs. Exercises such as resisted hip extension, single-leg bridges, and rotational core work (e.g., Pallof presses or dynamic planks) might help reinforce these key stabilizers, potentially enhancing lumbopelvic control during running.

It is important to interpret these results with caution, as several factors—such as adipose tissue, electrode positioning, and electrode movement—can influence surface EMG signals. Additionally, the use of different EMG acquisition methods (surface vs. intravaginal) between pelvic floor muscles and other muscles may affect signal comparability and interpretation.

Meanwhile, the relatively lower activation of the LE and PF muscles, likely a neuromuscular strategy to reduce fatigue and mechanical stress, emphasizes the importance of endurance-based and low-load training approaches for these areas. In clinical and training settings, this may translate to incorporating breathing-focused PF activation drills, low-load trunk extension work, or dynamic stability tasks that integrate the PF without over-recruitment. For runners with a history of lower back discomfort or pelvic floor dysfunction, this pattern might actually be protective, and training should aim to maintain this efficiency while ensuring baseline muscle function is preserved.

Furthermore, the weak to moderate correlation between BMI and lumbar activation in the MS group reinforces the need for individualized recommendations regarding footwear. Practitioners should consider anthropometric profiles when advising on shoe transitions, especially in women with a higher BMI, who may require a more gradual adaptation to avoid excessive compensatory loading.

Ultimately, while further research is needed to confirm these patterns, integrating these preliminary neuromuscular insights into screening, training, and rehabilitation protocols may contribute to more personalized and effective strategies for female runners.

## 6. Limitations

This study had several limitations that should be considered when interpreting the findings. The use of treadmill running may not fully replicate natural outdoor running biomechanics.

The short duration of the data collection at each speed (30 s) may not have been sufficient to capture muscle activation changes related to fatigue over time.

In addition, although participants did not report discomfort, the presence of the intravaginal EMG probe, comparable in size and shape to a tampon, may have subtly influenced the running biomechanics or muscle activation.

Finally, we did not assess the participants’ training histories, fitness levels, or previous sports experience, which could have influenced their neuromuscular activation patterns, particularly in the pelvic floor and core musculature. Additionally, foot posture (e.g., pronation, supination, or flat feet) was not assessed, which may have influenced individual responses to the footwear conditions.

It should also be considered that our results were obtained with the specified running shoes used in this study, so the results might not be generalized to other types of TSs or MSs with different characteristics.

## 7. Future Research Directions

Future research should aim to investigate muscle activation patterns in more heterogeneous and clinically relevant populations, particularly women presenting with urogenital dysfunctions such as stress urinary incontinence, in order to gain a greater understanding of neuromuscular adaptations influenced by footwear.

Longitudinal studies examining the incidence of running-related injuries and performance outcomes associated with different types of footwear are warranted to generate evidence-based guidelines for prevention and rehabilitation strategies.

In addition, assessing the effects of progressive habituation to minimalist footwear may provide information on the neuromuscular and biomechanical adaptations that occur over time.

## 8. Conclusions

No significant differences were found between minimalist and traditional shoes regarding EMG activity. Weak-to-moderate correlations emerged between the body mass index and the lumbar erector muscle activation with minimalist shoes, and between body mass index and the internal oblique with both footwear types. No significant correlations were found with pelvic floor muscles. The correlations between personal variables, shoe type, and muscle activation suggest that individual and external factors may influence neuromuscular modulation, impacting injury prevention and personalized interventions.

## Figures and Tables

**Table 1:** Median (P25, P75) of muscle activity (%MVIC) of the lumbar erector, gluteus maximus, internal oblique, and pelvic floor muscles at different running speeds (6, 9, and 11 km/h) and with two different footwear models (minimalist shoes and traditional shoes).

	6 km/h			9 km/h			11 km/h		
	Minimalist	Traditional	*p*	Minimalist	Traditional	*p*	Minimalist	Traditional	*p*
LE	9.06 ^a,b,c^ (5.46, 18.09)	10.57 ^a,b,c^ (6.00, 19.37)	0.666	8.88 ^a,b^ (5.78, 14.79)	10.20 ^a,b^ (5.12, 17.74)	0.157	8.35 ^a,b^ (5.31, 14.26)	11.19 ^a,b^ (5.10, 15.47)	0.253
GM	25.87 (14.59, 42.10)	21.89 (15.09, 43.07)	0.985	24.36 (14.87, 39.88)	24.18 (15.98, 41.32)	0.653	24.47 (10.47, 36.04)	23.63 (15.42, 36.90)	0.715
IO	33.40 (10.47, 52.51)	29.86 (12.99, 42.49)	0.646	37.42 ^c^ (14.23, 56.02)	34.43 ^c^ (18.58, 49.92)	0.800	37.70 ^c^ (16.30, 60.09)	36.69 ^c^ (20.71, 56.10)	0.708
PF	20.46 (7.14, 33.19)	18.44 (8.27, 35.49)	0.653	14.61 (5.38, 25.38)	14.28 (6.89, 26.80)	0.518	11.73 (5.90, 25.19)	12.00 (5.30, 27.15)	0.488
Intra-group comparison	*p* < 0.001	*p* < 0.001		*p* < 0.001	*p* < 0.001		*p* < 0.001	*p* < 0.001	

LE: lumbar erector, GM: gluteus maximus, IO: internal oblique, and PF: pelvic floor. Data are shown as medians (P_25_, P_75_). Intra-group comparison: comparisons of EMG activity between different muscles within the footwear group at each speed (Friedman test). Post hoc analysis: ^a^ *p* < 0.001 vs. GM, ^b^ *p* < 0.001 vs. IO, and ^c^ *p* < 0.001 vs. PF.

**Table 2:** Correlations between BMI and EMG activations of the different muscles at the three speeds used in this study when running with MSs and TSs.

	6 km/h		9 km/h		11 km/h	
	Minimalist	Traditional	Minimalist	Traditional	Minimalist	Traditional
**LE**	r = 0.463 **	r = 0.188	r = 0.426 *	r = 0.131	r = 0.439 **	r = 0.218
**GM**	r = 0.290 *	r = 0.228	r = 0.221	r = 0.235	r = 0.246	r = 0.235
**IO**	r = 0.397 *	r = 0.234	r = 0.402 *	r = 0.281 *	r = 0.438 **	r = 0.318 *
**PF**	r = −0.014	r = −0.022	r = −0.006	r = 0.010	r = 0.026	r = −0.022

LE: lumbar erector, GM: gluteus maximus, IO: internal oblique, PF: pelvic floor, and BMI: body mass index. * *p* < 0.05, ** *p* < 0.001.

## Data Availability

The data presented in this study are available upon request from the corresponding author. The data are not publicly available due to privacy and ethical restrictions.

## Abbreviations

The following abbreviations were used in this manuscript:

MSs	Minimalist shoes
TSs	Traditional shoes
IO	Internal oblique muscle
LE	Lumbar erector spinae muscle
PF	Pelvic floor
GM	Gluteus maximus muscle
EMG	Electromyographic
BMI	Body mass index
